# An Unexpected Cause of Syncope

**DOI:** 10.7759/cureus.38253

**Published:** 2023-04-28

**Authors:** Melisa Esposito, Sulagna Das, Yulith Roca Alvarez, Lyndi Schwartz

**Affiliations:** 1 Internal Medicine, Kettering Health, Dayton, USA; 2 Internal Medicine, Kettering Health Main Campus, Kettering, USA

**Keywords:** painless palpable mass, soft tissue sarcoma, dedifferentiated lipo, syncope, liposarcoma, retroperitoneal liposarcoma

## Abstract

Syncope is a common chief complaint among patients presenting to the emergency department, the etiology of which can often be discerned with a thorough history and physical examination. Inversely, liposarcomas are rare tumors that frequently pose a diagnostic challenge as the clinical presentation is highly nonspecific and varies greatly depending on the anatomic location and size of the tumor. Here we present a case of retroperitoneal liposarcomas (RLS) presenting to the emergency department (ED) with a sole complaint of syncope, resulting in a diagnostic dilemma. This clinical scenario highlights the significance of thorough physical examination regardless of the presenting chief complaint, as unexpected physical examination findings prompted an extended work-up and thus facilitated the diagnosis, providing the opportunity for early intervention and resection of the tumor.

## Introduction

Syncope comprises approximately 3% of emergency department (ED) visits, where up to 50% of cases can be diagnosed with a thorough history and physical examination [1]. Inversely, liposarcomas are rare tumors that often pose a diagnostic challenge as the clinical presentation is highly nonspecific and varies greatly depending on the anatomic location and size of the tumor [2,3]. When present in the retroperitoneum, liposarcomas often grow considerably before producing symptoms [2-4]. Consequently, patients may present with seemingly unrelated symptoms due to mass effect with resultant compression of nearby structures [2,3]. Given the poor prognosis and metastatic potential of liposarcomas, high clinical suspicion, and thorough physical evaluation are essential for early detection. Here we present a case of primary RLS presenting with a sole complaint of syncope, posing a diagnostic dilemma.

## Case presentation

**Figure 1:**
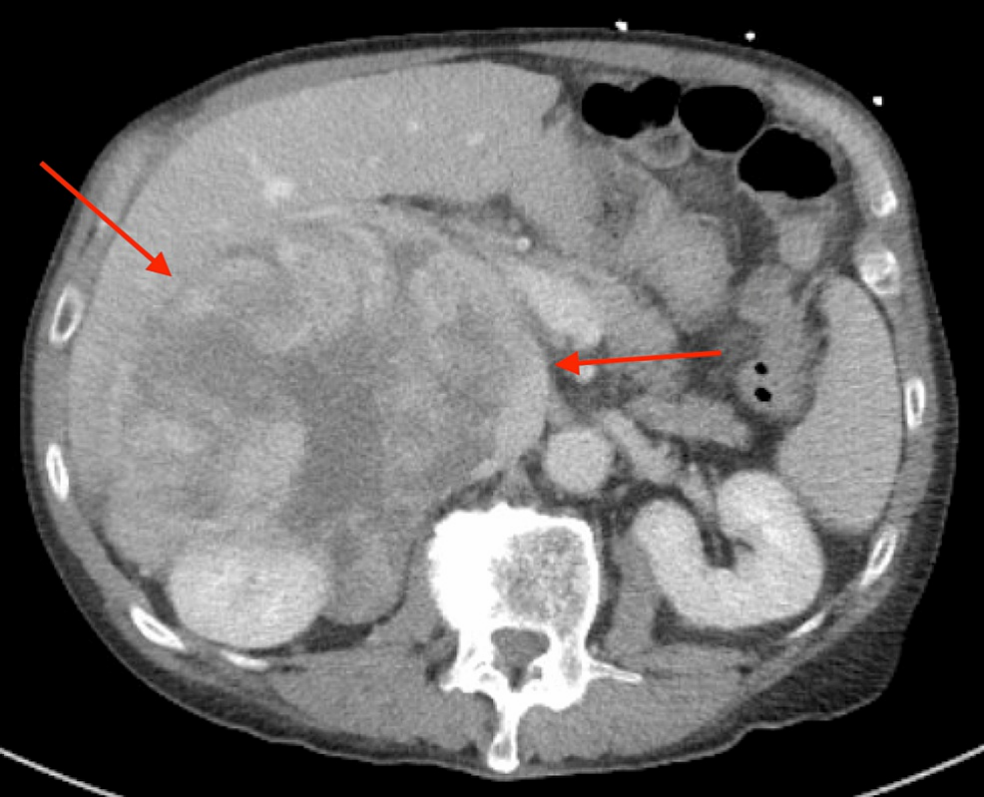
CTAP with intravenous contrast axial view revealing a large, heterogeneously enhancing retroperitoneal mass (red arrows).

**Figure 2:**
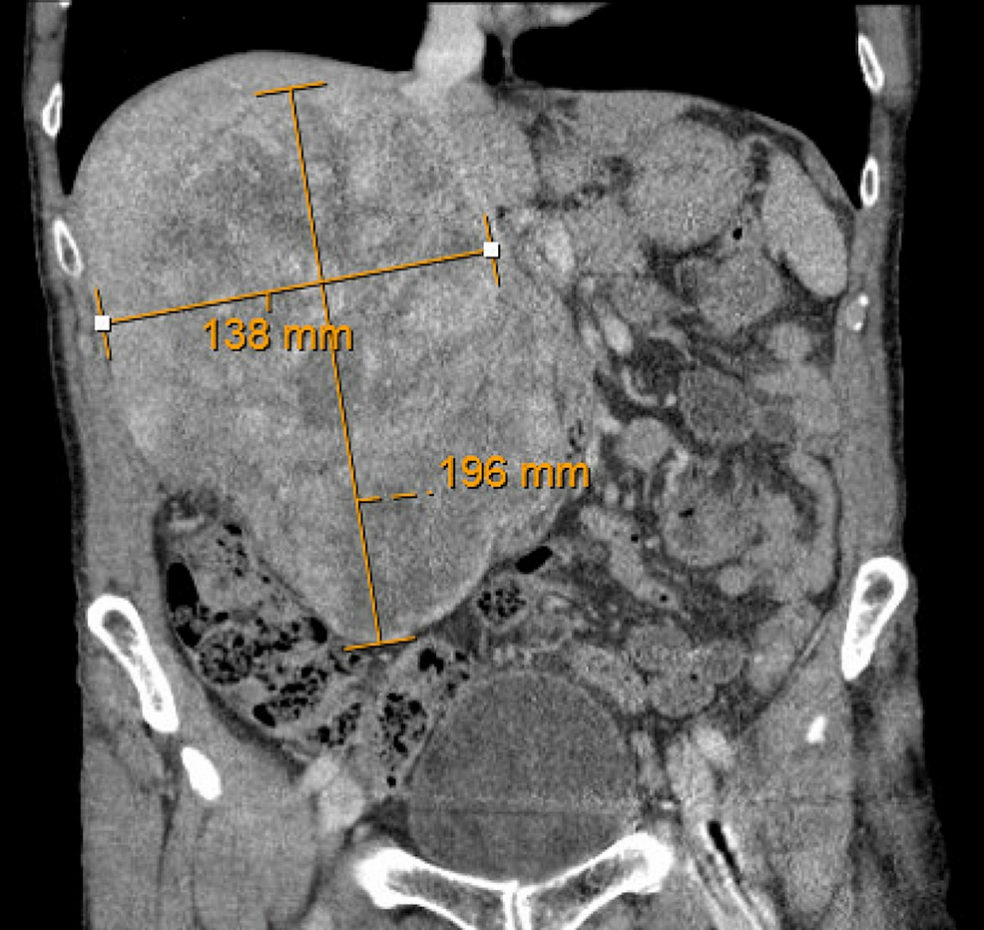
CTAP with intravenous contrast coronal view revealing a large, heterogeneously enhancing right retroperitoneal mass measuring approximately 19.6 x 13.8 x 11.2 cm in size.

A 75-year-old male with a past medical history of well-controlled hypertension and tobacco use disorder presented to the emergency department due to syncope. The patient described several occurrences of lightheadedness following positional changes and reported two episodes of collapse and loss of consciousness. He denied preceding chest pain, palpitations, shortness of breath, headache, blurry vision, paraesthesias, bowel or bladder incontinence, tongue biting, or other associated symptoms. Both episodes lasted a few seconds, and the patient reported an immediate return to baseline mentation after regaining consciousness. Vital signs were stable on presentation. The patient underwent extensive diagnostic evaluation, including comprehensive laboratory examination, 12-lead electrocardiogram and continuous cardiac monitoring, chest x-ray, computed tomography (CT) of the chest with angiography, and CT head without contrast, all of which was unrevealing. A large, indurated, palpable abdominal mass was noted in the right lower quadrant upon thorough physical examination. CT of the abdomen and pelvis with intravenous contrast was ordered and revealed a large 19.6 x 13.8 x 11.2 centimeter (cm) heterogeneous mass appearing to originate from the right adrenal gland (Figures 1-2).

**Figure 3:**
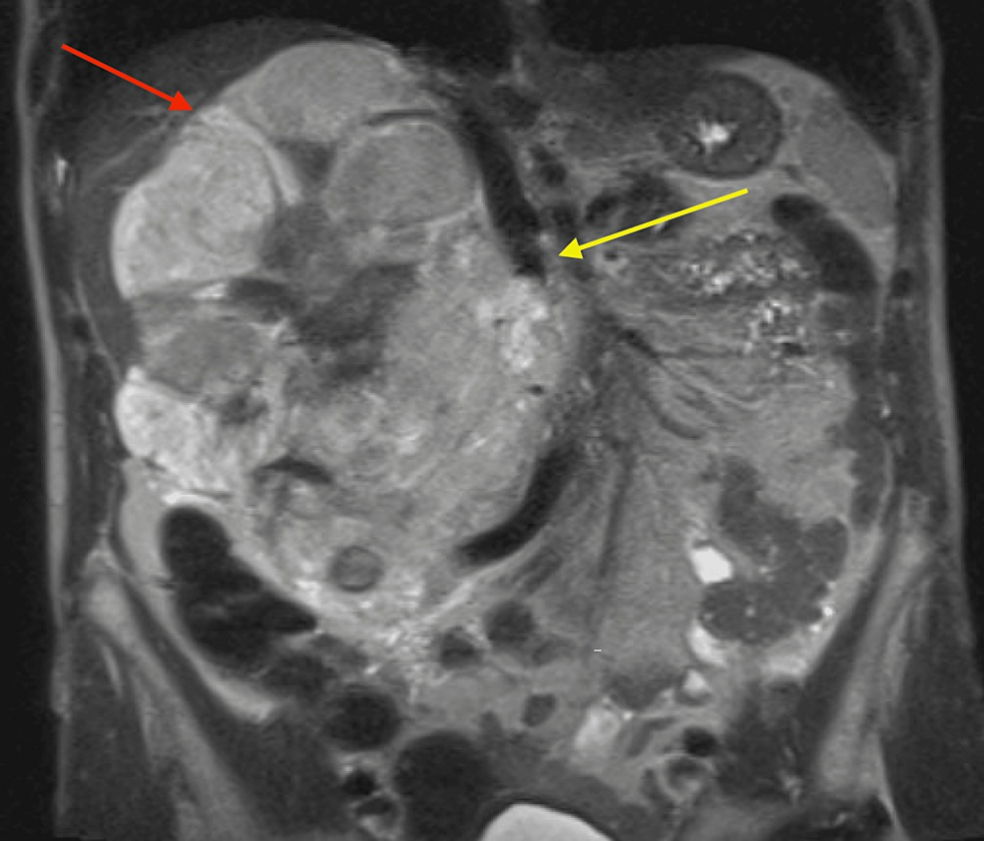
MRI adrenals with and without contrast coronal view revealing a large, T2 hyperintense heterogeneously enhancing mass (red arrow) in the right hemiabdomen, extending from right hemidiaphragm to right upper pelvis. Mass is seen compressing the inferior vena cava (yellow arrow).

**Figure 4:**
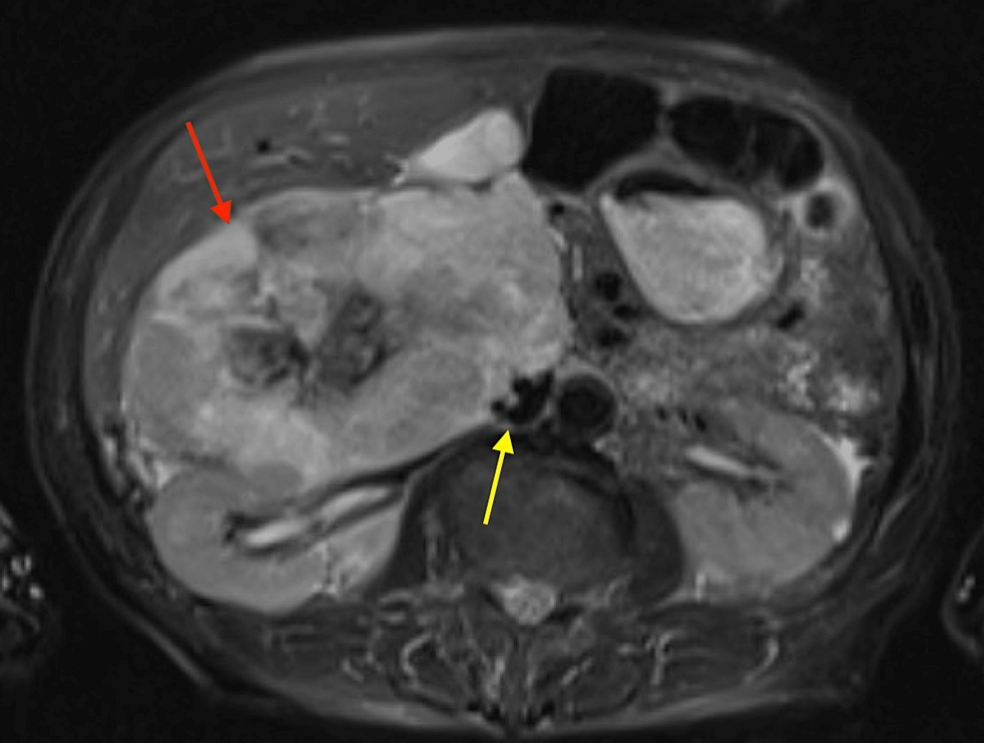
MRI adrenals with and without contrast axial view revealing a large, heterogeneously enhancing mass (red arrow) seen abutting and compressing the inferior vena cava (yellow arrow).

Subsequent evaluation with magnetic resonance imaging (MRI) of the adrenal glands with and without contrast further characterized a large, T2 hyperintense, heterogeneously enhancing mass in the right hemiabdomen extending from the right hemidiaphragm into the right upper hemipelvis, abutting the right kidney and right hepatic lobe. In addition, evidence of mass effect was noted with MRI revealing compression of the inferior vena cava (IVC) (Figures 3-4).

**Figure 5:**
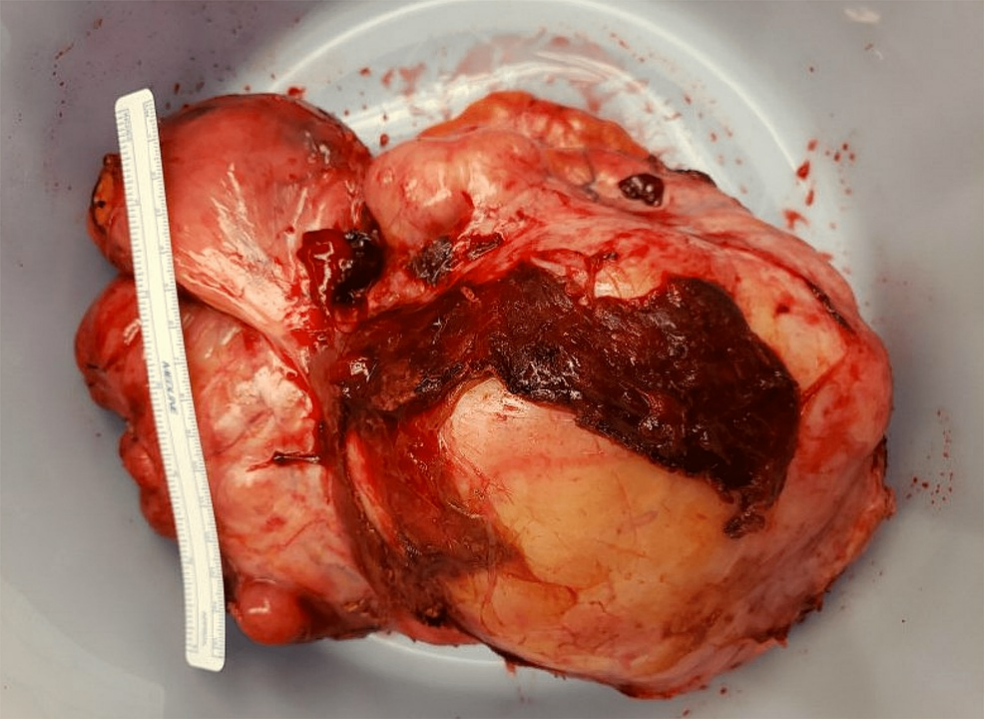
Photograph of large retroperitoneal liposarcoma status post surgical resection

It should be noted that the patient denied abdominal pain, distension, early satiety, or any other abdominal symptom, with his only complaints consisting of lightheadedness and syncope. Work-up was continued with functional biochemistry (dexamethasone suppression test, dehydroepiandrosterone sulfate, morning cortisol, renin, aldosterone/renin ratio, urine and plasma catecholamines, and urine and plasma metanephrines), which returned within normal limits. CT-guided biopsy of the mass initially showed a well-differentiated liposarcoma with amplification of the mouse double minute 2 (MDM2) gene at 12q15. Given the case's complexity, the patient was evaluated by surgical oncology, urology, and vascular surgery. Exploratory laparotomy revealed a large retroperitoneal mass enveloping the right kidney and adhering to the inferior edge of the liver, the duodenum, and the inferior vena cava. The patient ultimately underwent radical resection of a 24.6 cm right retroperitoneal mass with en bloc nephrectomy, adrenalectomy, partial right liver repair, and repair of the IVC (Figure 5).

Histopathologic examination of the intraoperative biopsy yielded a definitive diagnosis of high-grade dedifferentiated primary retroperitoneal liposarcoma (RLS) which was adherent to but did not invade the renal cortex, liver capsule, or adrenal gland. Margins were tumor-free, and the final staging was pT4N0M0, stage IIIB. Given no evidence of alternative etiology for the initial presentation, of dedifferentiated retroperitoneal liposarcoma was ultimately made due to a sole presenting symptom of syncope, caused by severe preload reduction from mass effect causing IVC compression.

## Discussion

Liposarcomas (LS) are rare soft tissue tumors typically occurring in middle-aged to older adults and are most often located in the retroperitoneum or extremities [5]. Several histologic subtypes of liposarcomas exist, including atypical lipomatous tumor/well-differentiated LS (WDL), dedifferentiated LS (DDL), and less common myxoid LS and pleomorphic LS [6]. Among RLS, dedifferentiated liposarcomas are most common, constituting approximately 45% of all retroperitoneal soft tissue sarcomas [7]. Though separate entities, WDL and DDL are thought to constitute a histologic and behavioral spectrum of one disease, with both subtypes associated with high-level amplifications in the chromosomal 12q13-15 region and exhibition of cyclin-dependent kinase 4 (CDK4) and MDM2 cell cycle oncogenes [5,8]. While WDL lacks metastatic capacity, up to 10-15% can dedifferentiate into DDL, increasing the risk of local recurrence and metastatic potential [2,5,8]. The clinical presentation of liposarcoma is nonspecific and varies depending on anatomic location. In RLS, common presenting symptoms include abdominal pain/distention, flank pain, early satiety, weight gain, increased abdominal girth, or gastrointestinal obstruction [2,3]. In addition, local invasion or compression of nearby structures may lead to seemingly unrelated symptoms, with neurologic, musculoskeletal, obstructive urinary/bowel symptoms, or, much less commonly, vascular symptoms [3]. When present in the retroperitoneum, given the large potential spaces of the retroperitoneum, liposarcomas often grow to considerable sizes before producing any symptoms, with the average liposarcoma measuring >20cm at the time of diagnosis [2-4]. Physical examination may be unremarkable or may reveal a palpable mass. Imaging is crucial for diagnosing RLS, with many cases detected incidentally after undergoing imaging ordered for an unrelated purpose. Contrast-enhanced CT and MRI are the preferred initial diagnostic studies for detecting primary sites and often yield clues as to histologic subtypes. Contrast-enhanced CT of the chest, abdomen, and pelvis is the staging investigation of choice [9].

Contrary to many other malignancies, as most liposarcomas are not F-Fluorodeoxyglucose (FDG) avid, positron emission tomography (PET)-CT scan is not routinely recommended as a component of diagnosis or staging [10-12]. Although imaging is an essential component of detection and surgical planning, retroperitoneal masses often pose a diagnostic challenge as anatomical distortion due to mass effect often makes it difficult to differentiate between the type of tumor and between primary (originating from the retroperitoneal space but not a retroperitoneal organ) and secondary (originating from a retroperitoneal organ) lesions [13]. Consequently, a histopathologic examination is the cornerstone of diagnosis, with immunohistochemical detection of cell cycle oncogenes CKD4 and MDM2, qualitative PCR, and FISH yielding a definitive diagnosis [5,8,13]. Surgical resection is the mainstay of treatment of nonmetastatic RLS. Given the considerable size at the time of detection and the frequent involvement of surrounding structures, complete macroscopic detection is technically challenging and often requires en-bloc removal of adjacent organs and soft tissue structures. The role of neoadjuvant and adjuvant chemotherapy and radiation in treating RLS is currently debated. The decision to incorporate these modalities depends on several factors, including but not limited to individual patient characteristics and preferences, provider experience, and facility guidelines. The overall prognosis of dedifferentiated liposarcoma is highly dependent on anatomic location, with RLS having a worse prognosis than elsewhere. RLS confers a local recurrence rate of approximately 40%, a metastatic rate of 15-30%, and overall disease-related mortality of 28% [14]. Curiously, mortality is most commonly due to uncontrolled local recurrence rather than distant metastasis. Given the potential for recurrence and metastasis, patients should be evaluated with serial imaging following surgical resection.

## Conclusions

Retroperitoneal liposarcomas are rare soft tissue tumors that often pose a diagnostic challenge due to nonspecific presenting symptoms. Given the poor prognosis and possible metastatic potential, a high clinical suspicion is essential for early diagnosis. This clinical scenario highlights the significance of thorough physical examination regardless of the presenting chief complaint, as unexpected physical examination findings prompted an extended work-up and thus facilitated the diagnosis, providing the opportunity for early intervention and resection of the tumor.
